# Meta-transcriptomic identification of *Trypanosoma* spp. in native wildlife species from Australia

**DOI:** 10.1186/s13071-020-04325-6

**Published:** 2020-09-05

**Authors:** Ayda Susana Ortiz-Baez, Kate Cousins, John-Sebastian Eden, Wei-Shan Chang, Erin Harvey, John H.-O. Pettersson, Scott Carver, Adam Polkinghorne, Jan Šlapeta, Karrie Rose, Edward C. Holmes

**Affiliations:** 1 Marie Bashir Institute for Infectious Diseases and Biosecurity, School of Life & Environmental Sciences and School of Medical Sciences, The University of Sydney, Sydney, NSW Australia; 2 Centre for Virus Research, Westmead Institute for Medical Research, Westmead, NSW Australia; 3 Zoonosis Science Center, Department of Medical Biochemistry and Microbiology, Uppsala University, Uppsala, Sweden; 4 Department of Biological Sciences, University of Tasmania, Hobart, TAS Australia; 5 Department of Microbiology and Infectious Diseases, NSW Health Pathology, Nepean Hospital, Penrith, NSW Australia; 6 The University of Sydney Medical School, Nepean Clinical School, Faculty of Medicine and Health, University of Sydney, Penrith, NSW Australia; 7 Laboratory of Veterinary Parasitology, Sydney School of Veterinary Science, The University of Sydney, Sydney, NSW Australia; 8 Australian Registry of Wildlife Health, Taronga Conservation Society Australia, Mosman, NSW Australia

**Keywords:** *Trypanosoma*, Australia, Native fauna, Genetic diversity, Meta-transcriptomics, sequencing

## Abstract

**Background:**

Wildlife species carry a remarkable diversity of trypanosomes. The detection of trypanosome infection in native Australian fauna is central to understanding their diversity and host-parasite associations. The implementation of total RNA sequencing (meta-transcriptomics) in trypanosome surveillance and diagnosis provides a powerful methodological approach to better understand the host species distribution of this important group of parasites.

**Methods:**

We implemented a meta-transcriptomic approach to detect trypanosomes in a variety of tissues (brain, liver, lung, skin, gonads) sampled from native Australian wildlife, comprising four marsupials (koala, *Phascolarctos cinereus*; southern brown bandicoot, *Isoodon obesulus*; swamp wallaby, *Wallabia bicolor*; bare-nosed wombat, *Vombatus ursinus*), one bird (regent honeyeater, *Anthochaera phrygia*) and one amphibian (eastern dwarf tree frog, *Litoria fallax*). Samples corresponded to both clinically healthy and diseased individuals. Sequencing reads were *de novo* assembled into contigs and annotated. The evolutionary relationships among the trypanosomatid sequences identified were determined through phylogenetic analysis of *18S* rRNA sequences.

**Results:**

We detected trypanosome sequences in all six species of vertebrates sampled, with positive samples in multiple organs and tissues confirmed by PCR. Phylogenetic analysis indicated that the trypanosomes infecting marsupials were related to those previously detected in placental and marsupial mammals, while the trypanosome in the regent honeyeater grouped with avian trypanosomes. In contrast, we provide the first evidence for a trypanosome in the eastern dwarf tree frog that was phylogenetically distinct from those described in other amphibians.

**Conclusions:**

To our knowledge, this is the first meta-transcriptomic analysis of trypanosomes in native Australian wildlife, expanding the known genetic diversity of these important parasites. We demonstrated that RNA sequencing is sufficiently sensitive to detect low numbers of *Trypanosoma* transcripts and from diverse hosts and tissues types, thereby representing an effective means to detect trypanosomes that are divergent in genome sequence.
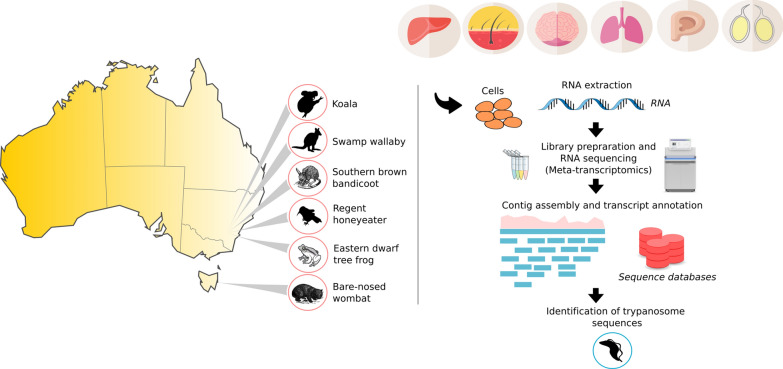

## Background

Trypanosomes are haemoprotozoan parasites that infect a wide range of animal taxa [1–3]. Endemic Australian fauna is a susceptible target for trypanosome infection, and several studies have revealed a remarkable diversity of trypanosomes in Australian wildlife [3–7]. This includes more than 15 species of exotic and endemic trypanosomes as well as several unclassified species [7, 8]. While some *Trypanosoma* species are associated with serious disease [9, 10], others play an undetermined role in the health of their hosts. For instance, the native trypanosomes *Trypanosoma copemani* and *T. vegrandis* have been associated with population declines of woylies (*Bettongia penicillata*) in Western Australia (WA) [9, 10]. It is likely that a similar phenomenon extends to other marsupial species, highlighting the need for continued surveillance [8, 11].

To date, most trypanosome surveillance has been directed toward screening Australian mammals (i.e. bats, marsupials, monotremes, and rodents). Marsupials, in particular, have been widely screened, allowing the identification of several trypanosome species (e.g. *T. copemani*, *T. irwini* and *T. gilletti*) [7, 11–13]. However, trypanosome infection has also been detected in other Australian vertebrate wildlife such as amphibians, birds, fish and reptiles [8, 14, 15]. Moreover, trypanosomes have been detected in hematophagous invertebrates that become infected while feeding on infected vertebrate hosts and which may act as parasite vectors [16, 17]. For example, in Australia, trypanosomes have been found in both aquatic leeches and ticks [5, 18, 19]. Other invertebrates such as lice, culicid mosquitoes, sand-flies, and tabanid flies are also believed to be potential trypanosome vectors [20–26]. However, because incidental infection during feeding is not necessarily associated with vector competence, further research is needed to determine the role of these hematophagous invertebrates in trypanosome transmission [8, 18].

Multiple trypanosome species have been documented in Australian wildlife. For example, surveillance in marsupials recorded up to five species (*T. irwini*, *T. gilletti*, *T. copemani*, *T. vegrandis* and *T. noyesi*) in koalas [4], with similar results in woylies and the southern brown bandicoot [9, 12]. In addition, the monitoring of Australian mammals has shown that *Trypanosoma* spp. are present in animals sampled on the east and west coasts of Australia, as well as Tasmania [7]. Despite this, there are clear gaps in sampling, and it is likely that trypanosomes are widespread across the Australian continent and in mammalian species [7].

Diagnosis of trypanosome infection largely relies on microscopy and a variety of molecular techniques [27]. PCR-based Sanger sequencing of genetic markers constitutes the gold standard for molecular diagnosis, including the *18S* rRNA gene in the small subunit rRNA (*SSU*), and the region encoding the glycosomal glyceraldehyde phosphate dehydrogenase (gGAPDH), an enzyme involved in the glycolytic pathway [28]. In recent years, a number of studies have implemented amplicon-based next-generation sequencing (NGS) to reveal the genetic diversity of trypanosomes in Australian marsupials [4, 6]. In comparison with conventional methods, NGS enables detection of trypanosome sequences at low copy number and target multiple genes with both high-throughput and accuracy. In addition, the development of meta-transcriptomics (i.e. bulk RNA sequencing) has enabled the detection and quantification of the transcripts expressed in the intra- and extracellular environments, including those derived from trypanosomes and other pathogens [29], and hence represents an increasingly valuable diagnostic tool [30–32].

Herein, we employed, for the first-time, a meta-transcriptomics approach as a method for the identification and surveillance of *Trypanosoma* in wildlife, screening different tissues from a variety of native Australian species. From this, we identified trypanosomes in several vertebrate groups from New South Wales (NSW) and Tasmania (TAS), including the identification of a divergent species of *Trypanosoma* in an amphibian species.

## Methods

### Sample collection

Most samples in this study were collected by the Australian Registry for Wildlife Health (ARWH) during monitoring surveys of wildlife, as well as from road-kill cases in NSW. The bare-nosed wombats were derived from road-kill in southern Tasmania. Following dissection, all tissue samples were stored at -80 °C until molecular analysis (Table 1). In total, we analysed 17 samples from different Australian native animal species, including four marsupials (koala, *Phascolarctos cinereus*; southern brown bandicoot, *Isoodon obesulus*; swamp wallaby, *Wallabia bicolor*; bare-nosed wombat, *Vombatus ursinus*), one bird (regent honeyeater, *Anthochaera phrygia*) and one amphibian (eastern dwarf tree frog, *Litoria fallax*). The amphibian specimen corresponded to a male diagnosed with severe, multisystemic, chronic trypanosomiasis (Additional file 1: Figure S1) and presumptive testicular *Myxobolus-*like infection. All individuals were identified to the lowest taxonomic level. Our sample set contained both healthy and diseased individuals (Table 1).

**Table 1:** Characterization of samples from Australian vertebrates that tested positive for trypanosome infection

Library	Host	No. of individuals examined	Tissue	Health status	Location	PCR result (*n*)
Vert1	Swamp wallaby (*Wallabia bicolor*) #1	1	Brain	Severe pulmonary congestion and oedema	Pittwater	Positive
Vert11	Regent honeyeater (*Anthochaera phrygia*)	1	Lung	Unknown	Sydney basin	Positive
Vert18	Bare-nosed wombat (*Vombatus ursinus*)	5	Liver	Healthy	Southern Tasmania	Positive (*n* = 3); negative (*n* = 2)
Vert21	Southern brown bandicoot (*Isoodon obesulus*)	1	Tail skin	Proliferative to ulcerative skin lesions	Sydney basin	Positive
Vert22	Koala (*Phascolarctos cinereus*)	7	Liver	Healthy	Sydney basin	Positive (*n* = 5); negative (*n* = 2)
Vert43	Eastern dwarf tree frog (*Litoria fallax*)	1	Testes and liver	Diseased	Kooragang island (NSW)	Positive
Vert48	Swamp wallaby (*Wallabia bicolor*) #2	1	Liver and ear	Lumpy jaw and ear lesions	Mimosa National Park (NSW)	Positive

*Note:* Libraries are indicated using alphanumeric codes and represent the collection of RNA fragments generated per sample for RNA sequencing

*Abbreviation*: n, number of samples

### Sample processing

In brief, total RNA was extracted from a variety of sample tissues (Table 1) using the RNeasy® Mini Kit (Qiagen) according to the manufacturer’s instructions. Sequencing libraries were generated using the TruSeq Stranded Total RNA Library Preparation protocol (Illumina) with host ribosomal RNA (rRNA) depletion (RiboZero Gold – Epidemiology). Subsequently, paired-end (100 bp) sequencing of the cDNA libraries was performed using the Illumina HiSeq 2000 system targeting at least 20M paired reads per library. All library preparation and sequencing were carried out by the Australian Genome Research Facility (AGRF).

### Meta-transcriptomic analysis

Sequence reads were trimmed for quality using the Trimmomatic tool [33] and assembled *de novo* into contigs using Trinity v. 2.5.1 [34] with default parameter settings. The relative abundance of transcripts was quantified as the number of transcripts per kilobase million (TPM). In short, this metric normalizes transcript abundance by transcript length and sequencing depth. For sequence identification, particularly of trypanosomes, the assembled contigs were compared against the NCBI GenBank nucleotide (nt) and non-redundant protein (nr) databases using BLASTN and DIAMOND v.0.9.32 [35] (Additional file 2: Table S1). Those contigs that exhibited matches to known trypanosome sequences with an e-value > 1 × 10^−70^ were retained for downstream analyses. Further, contigs corresponding to the stably expressed host mitochondrial marker, cytochrome *c* oxidase subunit 1 (*cox*1), were identified based on sequence alignments using DIAMOND. All contigs were aligned to reference sequences using BBMap v.37.98 and cross-validated to DIAMOND results to verify that the matches correspond to the vertebrate host. Abundance was quantified as the sum of relative abundances of contigs for the marker. Sequence contigs were annotated as follows: (i) to find conserved domains and classify protein families, sequences were compared against the Conserved Domain Database (CDD) [36] and InterProScan (http://www.ebi.ac.uk/interpro/); (ii) for gene assignment, all putative trypanosome contigs were aligned against a custom reference sequence database (genome assembly ASM21029v1) using DIAMOND [35].

### Confirmatory PCR

All samples included in this study were screened for *Trypanosoma* infection *via* PCR assays using primers targeting 2136-bp (outer) and 320-bp (nested) fragments of the *18S* rRNA (Additional file 3: Table S2). In general, the cDNA was synthesised from up to 100 ng of total RNA using random hexamers and SuperScript™ VILO™ (Invitrogen, CA, USA). The RT-PCR reactions proceeded as follows: 10 min of random priming at 25 °C, 20 min of extension at 50 °C, and 5 min of RT denaturation at 85 °C. Similarly, the PCR reactions with Platinum™ SuperFi™ (Invitrogen) were performed as follows: 1 min of hot start at 98 °C, followed by 40 cycles consisting of denaturation at 98 °C for 10 s, primer annealing for 10 s, and then extension at 72 °C according to conditions described in Additional file 3: Table S2. A final elongation step was run at 72 °C for 1 min. PCR products were visualized by electrophoresis on a 1.5% agarose gel stained with ethidium bromide. Controls were included to identify potential cross-contamination in reagents.

### Phylogenetic analysis

The trypanosome contigs obtained here were compared with homologous sequences retrieved from GenBank, using *18S* rRNA as a key phylogenetic marker (Additional file 4: Table S3). Multiple sequence alignment (n = 81) was conducted using the E-INS-i algorithm in MAFFT v7.450. The best-fit model of nucleotide substitution (i.e. GTR+F+I+Γ_4_) was determined by using the Akaike information criterion (AIC) in the ModelFinder program [37] implemented in IQ-TREE v1.6.7 [38]. Phylogenetic relationships were then inferred using the maximum likelihood method [39] available in IQ-TREE v1.6.7 [38]. Nodal support values were also assessed by using a SH-like approximate Likelihood Ratio Test (SH-aLRT) and 1000 ultrafast bootstrap (UFBoot) replicates [40].

## Results

### Detection of *Trypanosoma* in screened samples

Using a meta-transcriptomic approach, we successfully identified trypanosome transcripts in six Australian species sampled in NSW and TAS, corresponding to the animal classes Amphibia, Aves and Mammalia. Trypanosome transcripts were detected in 60% (3 out of 5) of bare-nosed wombats, 71.43% (5 out of 7) of koalas, in both of the swamp wallaby samples, reagent honeyeater (*n* = 1), southern brown bandicoot tail (*n* = 1), and the eastern dwarf tree frog (*n* = 1). In total, trypanosomes were detected in 76.47% (13/17) of the individuals screened. With respect to target tissues, we detected trypanosome transcripts across a variety of tissues in infected individuals (Table 1), and positive samples were collected from both apparently healthy and diseased individual animals.

Despite the widespread presence of *Trypanosoma* in the samples characterized, we observed marked variation in the abundance and number of *de novo* assembled contigs among libraries. In general, the host *cox*1 transcripts were ~ 60% to ~ 99% more abundant than trypanosome transcripts (Table 2). Since samples showing high abundance of host *cox*1 also exhibited variable levels of abundance for trypanosome transcripts, these results suggest that the variation in abundance levels among samples was not due to biases in sampling processing. In addition, most transcripts were detected in the swamp wallaby #2 sample (*n* = 314, i.e. 0.05% of total transcripts per library) followed by the eastern dwarf tree frog (*n* = 149, i.e. 0.03% of total transcripts per library), whereas the lowest number of transcripts was identified in the regent honeyeater (*n* = 3, i.e. 0.0008% of total transcripts per library) (Table 3; Additional file 2: Table S1). Top BLAST hits ranged from 241 bp to 2258 bp, targeting regions corresponding to the transcribed spacers (ITS1, ITS2) and the *5.8S* rRNA, *18S* rRNA and *28S* rRNA of the large subunit of the ribosome. Similarly, we recovered hits against uncharacterized proteins, the surface protease GP63, and the heat shock proteins (HSPs) of *Trypanosoma*.

**Table 2:** Contigs with Blast hits to the small subunit (SSU) *18S* rRNA in the nt/nr database

Host	Contig accession	Length	TPM Tryp	e-value	Hit	Gene *SSU*	TPM *cox*1
Swamp wallaby (*Wallabia bicolor*) #1	VERT1_DN159759_c0_g1_i1*	299	3.27	9E−152	*Trypanosoma* sp. TL.AQ.22	*18S* rRNA	30192.26
	VERT1_DN215626_c0_g1_i1*	318	3.06	3E−162	*Trypanosoma* sp. TL.AQ.45	*18S* rRNA	
Regent honeyeater (*Anthochaera phrygia*)	VERT11_DN10127_c0_g1_i1*	666	2.82	0.00E+00	*Trypanosoma thomasbancrofti*	*18S* rRNA	512.02
Bare-nosed wombat (*Vombatus ursinus*)	VERT18_DN14693_c0_g1_i1*	615	2.94	0.00E+00	*Trypanosoma* sp.	*18S* rRNA	3805.74
	VERT18_DN33207_c0_g1_i1	241	3.42	1E−118	*Trypanosoma* sp. AB-2013	*18S* rRNA	
	VERT18_DN9224_c0_g1_i1	491	1.87	0.00E+00	*Trypanosoma* sp.	*18S* rRNA	
Southern brown bandicoot (*Isoodon obesulus*)	VERT21_DN254377_c0_g1_i1*	411	0.64	0.00E+00	*Trypanosoma* sp. LM-2010	*18S* rRNA	577.36
Koala (*Phascolarctos cinereus*)	VERT22_DN394953_c0_g1_i1*	241	0.86	3E−118	*Trypanosoma irwini*	*18S* rRNA	2622.98
Eastern dwarf tree frog (*Litoria fallax*)	VERT43_DN68004_c3_g3_i2*	1728	46.71	0.00E+00	*Trypanosoma* sp. 858	*18S* rRNA	1258.51
Swamp wallaby (*Wallabia bicolor*) #2	VERT48_DN150018_c0_g6_i1	718	55.01	0.00E+00	*Trypanosoma pestanai* LEM 110	*18S* rRNA	2152.22
	VERT48_DN190740_c0_g1_i1*	433	3.59	0.00E+00	*Trypanosoma* sp. H26	*18S* rRNA	
	VERT48_DN367248_c0_g1_i1*	890	743.4	0.00E+00	*Trypanosoma* sp. LM-2010	*18S* rRNA	

* Contigs used for phylogenetic analysis based on the composition chi-square test performed by IQ-TREE

**Table 3:** Summary of top *Trypanosoma* hits from BLAST in the nt/nr database

Host	No. of contigs with hits for *Trypanosoma*	Length of best hit contig	Best BLAST hits against nr (DIAMOND)	Region	Best hit e-value	Gene	Best BLAST hits against nt/nr	Best hit e-value	Region
Swamp wallaby (*Wallabia bicolor*) #1	8	513	*T. theileri*	Uncharacterized protein	6.10E−49	TM35_000063140	*T. minasense*	0.00E+00	*18S* rRNA, ITS1, *5.8S* rRNA, ITS2, *28S* rRNA
Regent honeyeater (*Anthochaera phrygia*)	3	421	*T. theileri*	Uncharacterized protein	5.50E−48	TM35_000063130	*T. minasense*	0.00E+00	*18S* rRNA, ITS1, *5.8S* rRNA, ITS2, *28S* rRNA,
Bare-nosed wombat (*Vombatus ursinus*)	5	539	*T. theileri*	Uncharacterized protein	9.30E−32	TM35_000063140	*T. pestanai*	0.00E+00	*28S* rRNA
Southern brown bandicoot (*Isoodon obesulus*)	7	703	*T. theileri*	Uncharacterized protein	7.30E−37	TM35_000063140	*T. rangeli*	0.00E+00	*28S* rRNA
Koala (*Phascolarctos cinereus*)	24	241	*T. theileri*	Uncharacterized protein	5.80E−34	TM35_000063130	*T. theileri*	4E−106	Uncharacterized protein
Eastern dwarf tree frog (*Litoria fallax*)	149	1267	*T. cruzi*	Heat-shock protein 85, putative, partial	1.80E−195	Tco025E_09708	*T. conorhini*	0.00E+00	Heat-shock protein 90
Swamp wallaby (*Wallabia bicolor*) #2	314	2258	*T. cruzi*	PWU95505.1 putative surface protease GP63	1.7E-143	TM35_000063130	*Trypanosoma grayi* surface protease GP63 partial mRNA	1E−60	Surface protease GP63

To place trypanosome sequences into a phylogenetic context (see below), and hence achieve taxonomic assignment, we identified the contigs targeting the *18S* rRNA of the SSU. Abundance levels of *18S* rRNA contigs ranged from 0.64 to 743.40 TPM. The highest cumulative abundances were identified in the eastern dwarf tree frog (TPM = 46.71) and the swamp wallaby #2 (TPM = 802) (Table 2), while the Southern brown bandicoot showed the lowest values (TPM = 0.64). In comparison, the host reference gene *cox*1 was abundantly expressed across samples (TPM: 512.02–30,192.26), with the highest levels observed in the swamp wallaby #1 sample (TPM = 30,192.26).

To validate these results, we used PCR assays and generic primers targeting the *18S* rRNA gene (Additional file 3: Table S2) to detect trypanosome infection in all samples analyzed. Samples comprised a number of organs and tissues, including brain (*n* = 1), ear (*n* = 1), liver (*n* = 14), lung (*n* = 1), tail (*n* = 1), and testes (*n* = 1). A 320-bp nested fragment corresponding to the *18S* rRNA was amplified in all samples containing trypanosomes, as previously identified by meta-transcriptomics (Table 1).

### Phylogenetic analysis of *Trypanosoma*-positive samples

**Fig. 1:**
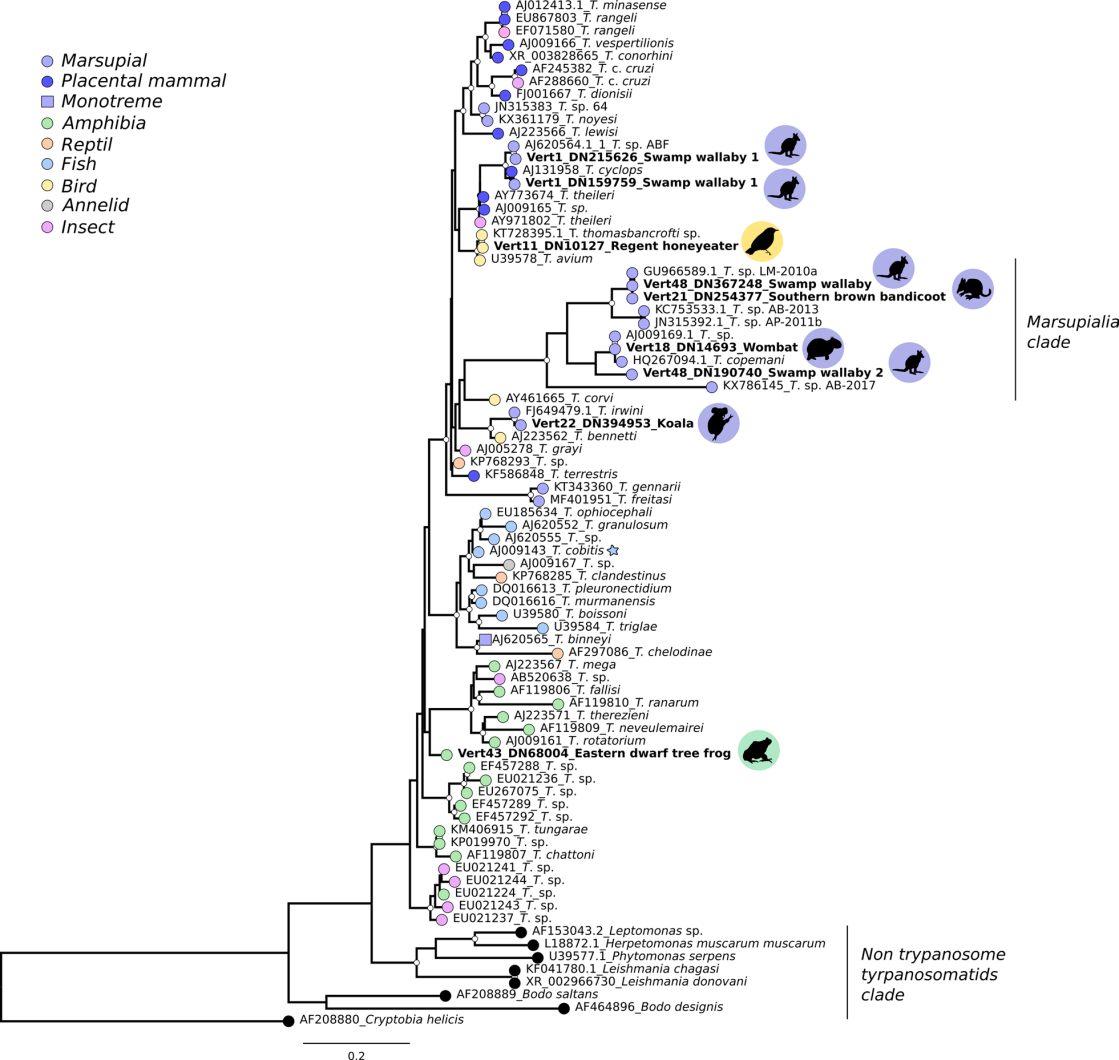
Maximum likelihood phylogenetic tree depicting the evolutionary relationships among trypanosomes sampled here (branch labels in bold) and background representative sequences. Branch tips are colored according to the host of sampling. Trypanosomes detected in fish and annelids are indicated by a star. Animal silhouettes represent the hosts that tested positive for trypanosome infection. Node support values (SH-aLRT > 80% and UFBoot > 95%) are indicated with white circle node shapes in the tree. *Trypanosoma* sp. ABF was also identified in a specimen from NSW

Phylogenetic analysis revealed that trypanosomes infecting the Australian native species covered in our study were generally closely related to known trypanosome species (Fig. 1). We identified trypanosome sequences in the specimens of the swamp wallaby that fell into two separate clades associated with placental and marsupial mammals. However, most samples grouped with different trypanosomes identified from marsupials, forming a group that we term the “Marsupialia” clade (Fig. 1**)**. This clade can be further divided into two groups: the first includes trypanosomes from the wallaby and the southern brown bandicoot, while the second group contained trypanosomes from the wallaby and bare-nosed wombat. Strikingly, the trypanosome from the koala fell into a different clade that is related to *T. gennarii* (nucleotide sequence similarity of 81.30%) and *T. freitassi* (82.04%) identified in South American marsupials (*Monodelphis* spp.), *T. bennetti* (92.56%) in birds (*Falco sparverius*) and *T. irwini* (98.75%) in koalas. Moreover, we identified a trypanosome species in the regent honeyeater that is closely related to the avian trypanosomes *T. thomasbancrofti* and *T. avium* that share ~100% and 97% sequence similarity, respectively. Sequence comparisons against avian genotypes 1–4 (classification *sensu* Šlapeta et al. (2016) [41]) showed a perfect match with genotype 1 of *T. thomasbancrofti* (Additional file 5: Table S4), indicating that the regent honeyeater trypanosome likely belongs to that species.

In addition to the trypanosomes related to mammals and birds, we identified a trypanosome species infecting the eastern dwarf tree frog that was divergent from other trypanosomes in amphibians (Additional file 1: Figure S1). Notably, this amphibian trypanosome was related to those present in other amphibians, reptile and insect species, although it fell in a phylogenetically divergent position in the clade (with relatively strong support; SH-aLRT 89.6%; UFBoot 76%) and hence represents a novel lineage. The position of the dwarf tree frog sequence remained unchanged following additional analyses including a broader range of fish, reptile and leech transcriptomes (data from ref. [16]), indicating that it is not an artefact due to biases in taxon sampling (Additional file 6: Figure S2).

## Discussion

We have, to our knowledge for the first time, implemented a meta-transcriptomic approach for detecting *Trypanosoma* spp., investigating a variety of wildlife species indigenous to Australia. Unlike conventional methods for trypanosome diagnosis (cellular culture, PCR assays, and Sanger sequencing) [42], meta-transcriptomics represents an unbiased approach for the detection of parasite diversity within samples, only requiring sufficient levels of gene expression [29]. To date, only a few surveillance studies have applied NGS technologies for the detection of trypanosomes in wildlife, although this approach is able to identify mixed trypanosome infections in marsupials and effectively screen their ectoparasites [4, 6]. Using total RNA sequencing we identified trypanosomes in four marsupials, one bird and one amphibian species, highlighting the ability of this approach to detect parasites in a range of host species and target tissues (Table 1). Hence, meta-transcriptomics enables the detection of trypanosomes in a broad range of samples that might include symptomatic and subclinical infections, different stages of disease, as well as variable levels of parasitemia.

Most of the trypanosome transcripts identified in the hosts analyzed were associated with genes encoding ribosomal components, suggesting that ribosome biogenesis and protein synthesis have a central role in the infection process (Tables 2, 3). In the case of the heat-shock protein 90 (Hsp90) identified in the eastern dwarf tree frog, the presence of this molecular chaperone has been associated with transitions across trypanosome life-cycle stages [43]. Hsp90 synthesis induction has also been related to stress responses in *T. cruzi*, reflecting the change in temperature when the parasite moves from the vector to the mammalian host [44, 45]. Hsp90 is also known to play an essential role in protein folding and degradation under normal conditions [46, 47]. The major surface protease GP63 identified in swamp wallaby #2 is a highly immunogenic antigen involved in macrophage-parasite interaction encoded by a multi-copy gene that also occurs in *Leishmania* [48, 49]. Differential expression of GP63 is associated with the parasite life-cycle, with genetic variation facilitating immune evasion and colonization [48, 50].

Previous studies have suggested that trypanosomes often have deleterious effects on the health of the infected hosts [9–11, 51, 52]. As the trypanosomes described here were detected in both healthy and diseased individuals, we are unable to make inferences on their capacity to cause disease (Table 1). Indeed, many of the health conditions manifest in the animals studied were unspecific or prone to be associated with other sort of infections. For instance, the pulmonary congestion and oedema in the swamp wallaby #1 sample may be consistent with orbivirus infection symptoms (family *Reoviridae*) [53], while the pox-like lesions in the southern brown bandicoot have been previously associated with infection by the Bandicoot papillomatosis and carcinomatosis virus (BPCV2) (*Polyomaviridae*) in the western barred bandicoot (*Perameles bougainville*) [54]. Similarly, although the ear lesions in the swamp wallaby #2 could be attributed to trypanosome infection, other causative pathogens could be associated with the lumpy jaw and emaciation [55, 56]. In addition, the eastern dwarf tree frog was co-infected with *Trypanosoma* and *Myxobolus*, confounding the association of disease with any etiological agent. Because our study was limited to vertebrates, it does not provide insights into the potential vector involved in parasite transmission. However, as suggested in previous studies, it is possible that both ticks and dipterans (i.e. flies and mosquitoes) are vectors of these parasites as they can feed on a large variety of hosts including mammals, birds and amphibians [4, 18–20, 22, 57, 58]. Some hemipterans might also play a vectorial role in the transmission of trypanosomes in sylvatic and peridomestic settings, as documented in the Americas [59–61]. Clearly, more research is needed to clarify the vectors and the mode of trypanosome transmission in Australian wildlife [8, 18, 19].

Phylogenetic analysis revealed that the trypanosomes identified in native Australian fauna fell into different lineages that were largely concordant with that of the host species from which they were sampled, although we were unable to make taxonomic assignments to the species level. Notably, we identified three distinct clades of marsupial trypanosomes (Fig. 1). The trypanosome species detected in the swamp wallaby that fell outside the Marsupialia clade was closely related to *Trypanosoma* sp. ABF previously described in the swamp wallaby in NSW [8], and to *T. cyclops*, an exotic trypanosome isolated from the monkey *Macaca nemestrina* and related to *T. theileri*-related trypanosomes in ruminants and tabanids. The relatedness among these trypanosome species raises concerns over the potential susceptibility of Australian vectors and vertebrates to infection by exotic trypanosomes and hence the establishment of a zoonotic transmission cycle [7, 8]. In addition, although most marsupial trypanosomes analyzed fell into the Australian Marsupialia clade, trypanosome species infecting these mammals did not form a monophyletic group, indicative of a history of cross-species transmission [62].

Among the trypanosome species infecting marsupials, *T. irwini*, *T. gilletti*, *T. copemani*, *T. vegrandis*, *T. noyesi* and *Trypanosoma* sp. AB-2017 have been described in koalas [4, 7, 13]. Our results indicated that *Trypanosoma* sp. detected in the koala was closely related to *T. irwini* and the avian exotic trypanosome *T. bennetti.* Given than the former has been also identified in koalas, the trypanosome detected in the sampled koala likely corresponds to *T. irwini*. The close relationship between the *T. irwini* and *T. bennetti* has been previously documented [8, 13] and is compatible with the hypothesis that hosts sharing similar environments and vectors are susceptible to related parasites (i.e. “host-fitting”) [8, 63]. This provides an explanation for the relationship between trypanosomes infecting arboreal fauna inhabiting distant regions.

The trypanosome sequence we identified in the regent honeyeater likely belongs to *T. thomasbancrofti* (genotype 1), and *T. thomasbancrofti* was originally described in the regent honeyeater [41]. This trypanosome species has been suggested to be a culicid-vectored parasite and has been detected in healthy captive and wild regent honeyeaters [41]. In contrast, *T. avium* was identified in the rook (*Corvus frugilegus*) and associated with serious disease and death in birds, with suggestions that it is transmitted by blackflies (*Simulium* spp.) [64, 65] and phlebotomine sand flies [21]. Hence, our data corroborated the presence of *T. thomasbancrofti* in the regent honeyeater and highlight the importance of parasitological surveillance in the wild for this species classified as critically endangered (CR) (*sensu* IUCN).

Of particular interest was the case of the trypanosome detected in the eastern dwarf tree frog that was related to those identified in amphibians, reptile, and insect species. Since this amphibian trypanosome fell in a divergent and basal position within the clade it might represent a new trypanosome species and hence merits further characterization (Additional file 1: Figure S1; Additional file 6: Figure S2). Interestingly, considering the clinical diagnosis of the frog sampled (see Methods) as well as its transcript abundance (Table 3), it is possible that this trypanosome species or the synergistic infection by *Trypanosoma* with *Myxobolus* might have detrimental effects on amphibian health. This clearly merits further investigation. To our knowledge, this is the first report of a trypanosome in the eastern dwarf tree frog (Additional file 1: Figure S1), although amphibians are known to be parasitized by different trypanosomes species [15, 16, 66–68] and some have been documented in Australian amphibians [15, 67, 69]. That the clade containing the eastern dwarf tree frog sequence also contains a trypanosome infecting sand flies tentatively suggests that dipterans or other invertebrates could play a role vectoring trypanosome transmission [58].

While our study was focused on samples collected from multiple organs and tissues, meta-transcriptomics has previously been shown to be an efficient approach for characterizing blood parasites, even at low abundance [29, 70]. In addition, the technique has been used to detect trypanosome sequences in the blood meals of *Ixodes holocyclus* ticks and *Aedes camptorhynchus* mosquitoes [19, 71]. Hence, when combined with more traditional approaches, meta-transcriptomics offers a promising way to shed new light on the ecology and epidemiological surveillance of parasites in nature, although the approach is costly, requires extensive computational resources and may be unable to detect genes that are not expressed to sufficient levels [70].

## Conclusions

To our knowledge, this is the first meta-transcriptomic analysis of trypanosomes in native Australian wildlife, expanding the known genetic diversity of these important parasites. Our findings highlight the diversity of trypanosomes infecting an important spectrum of Australian native fauna. We also demonstrated that RNA sequencing is sufficiently sensitive to detect low levels of *Trypanosoma* transcripts from diverse hosts and tissues types, and hence represents an effective means to detect trypanosomes that are divergent in genome sequence.

## Supplementary information

**Additional file 1: Figure S1.** Light microphotograph of the promastigote phase of *Trypanosoma* sp. in giemsa-stained blood film from *Litoria fallax.* Scale bar represents 10 μm.

**Additional file 2**: **Table S1.** Summary of contigs with hits against trypanosome sequences at the NCBI nucleotide (nt) database. Relative abundance was calculated for each contig as transcripts per million (TPM).

**Additional file 3: Table S2.** List of PCR primers used in this study for confirmation of trypanosome infection.

**Additional file 4: Table S3.** List of sequences used for phylogenetic analysis.

**Additional file 5: Table S4.** Pairwise sequence identity among *18S* rRNA sequences of avian trypanosomes belonging to genotypes 1–4 and the putative *T. thomasbancrofti* identified in this study. Genotype classification *sensu* Šlapeta et al. (2016) [41].

**Additional file 6: Figure S2.** Maximum likelihood tree showing phylogenetic relationships among trypanosomes within the aquatic clade based on the *18S* rRNA gene. The trypanosome identified in *Litoria fallax* is indicated in blue. The hosts of trypanosomes are indicated with colour-coded tips.

## Data Availability

All data generated or analysed during this study are included in this published article and its additional information files. The newly generated contig sequences were deposited in the GenBank database under the accession numbers MT732373-MT732384. All new sequence reads are available at the NCBI Sequence Read Archive (SRA) database under the BioProject accession PRJNA626677 (BioSample accessions: SAMN15401543 - SAMN1540159). The dataset supporting the conclusions of this article is available in the figshare repository, https://figshare.com/s/d9c281ada61d8a8ed884.

## Abbreviations

BLAST: Basic Local Alignment Search Tool
BPCV2: bandicoot papillomatosis carcinomatosis virus type 2
*cox*1: cytochrome *c* oxidase subunit 1
CR: critically endangered
gGAPDH: glycosomal glyceraldehyde phosphate dehydrogenase
GP63: major surface glycoprotein
HSPs: heat-shock proteins
IUCN: International Union for Conservation of Nature
NCBI: National Center for Biotechnology Information
NSW: New South Wales
nr: non-redundant database
nt: nucleotide database
PCR: polymerase chain reaction
rRNA: ribosomal ribonucleic acid
RT-PCR: reverse transcription polymerase chain reaction
SSU: small subunit
TAS: Tasmania
Tryp: trypanosome
TPM: transcripts per million
