# Supplementary figures and images for: Meta-transcriptomic identification of Trypanosoma spp. in native wildlife species from Australia
Source: Parasit Vectors. 2020 Sep 5;13:447. doi: 10.1186/s13071-020-04325-6 (PMC7487544; doi:10.1186/s13071-020-04325-6)

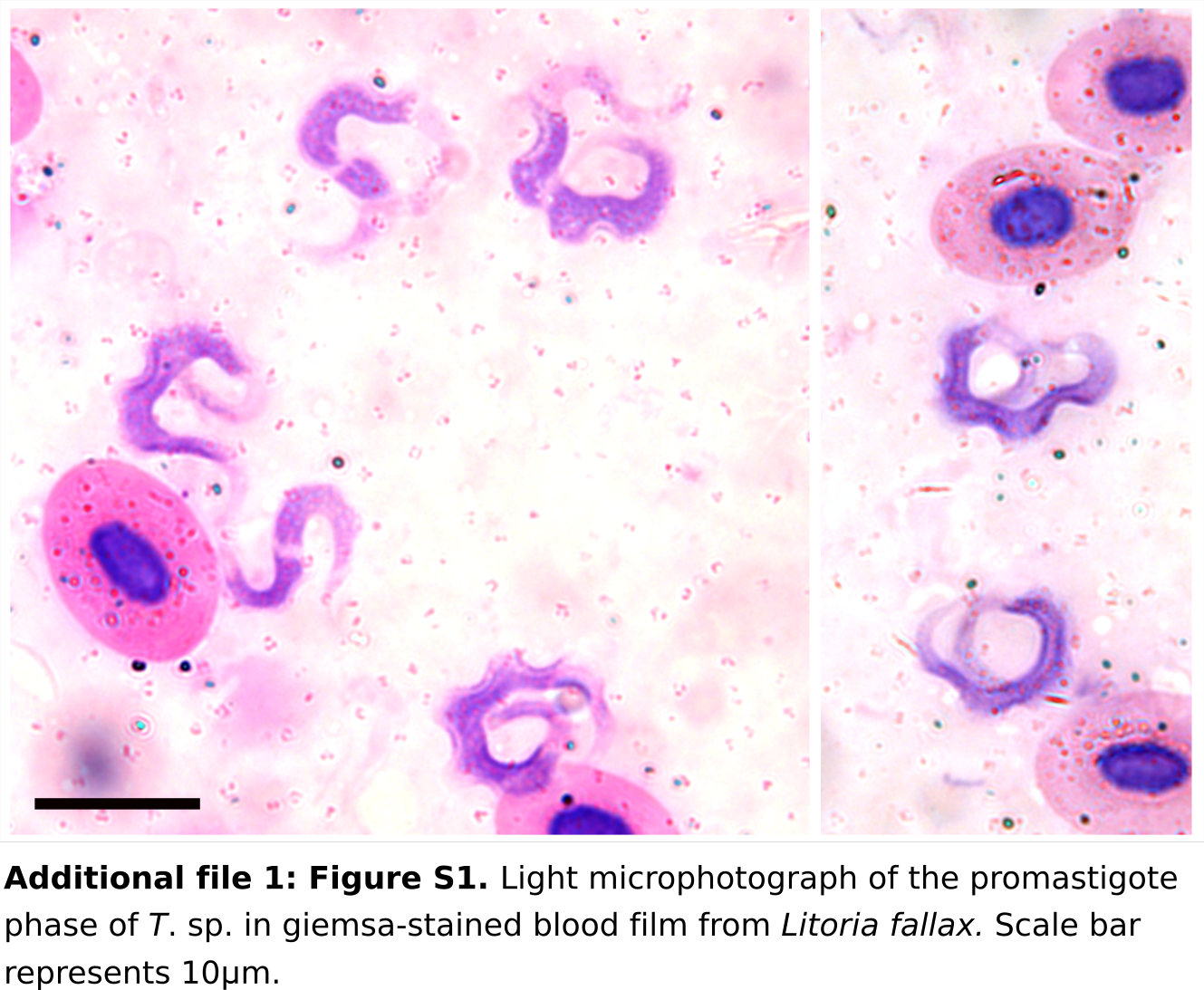

Supplement: Supplementary file 1 — Additional file 1: Figure S1. Light microphotograph of the promastigote phase of Trypanosoma sp. in giemsa-stained blood film from Litoria fallax. Scale bar represents 10 μm. [file 13071_2020_4325_MOESM1_ESM.tif]
