# Supplementary material for: Meta-transcriptomic identification of Trypanosoma spp. in native wildlife species from Australia
Source: Parasit Vectors. 2020 Sep 5;13:447. doi: 10.1186/s13071-020-04325-6 (PMC7487544; doi:10.1186/s13071-020-04325-6)
Supplement: Supplementary file 3 — Additional file 3: Table S2. List of PCR primers used in this study for confirmation of trypanosome infection. [file 13071_2020_4325_MOESM3_ESM.docx]

| Description | Primer ID | Direction | Sequence | Target (bp) | Author | Annealing (°C) | Elongation (min:sec) |
| --- | --- | --- | --- | --- | --- | --- | --- |
| Vertebrate *Trypanosoma* 18S (nested) | S755 | Forward | CTACGAACCCTTTAACAGCA | 320 | Maslov *et. al* 1996 [65] | 60.7 | 0:15 |
|  | S823 | Reverse | CGAAYAACGCYCTATCAGC |  |  |  |  |
| Vertebrate *Trypanosoma* 18S (outer) | S762 | Forward | GACTTTTGCTTCCTCTAWTG | 2136 | Maslov *et. al* 1996 [65] | 61.4 | 1:30 |
|  | S763 | Reverse | CATATGCTTGTTTCAAGGAC |  |  |  |  |

**Additional file 3: Table S2.** List of PCR primers used in this study for confirmation of trypanosome infection.
