# Supplementary material for: Meta-transcriptomic identification of Trypanosoma spp. in native wildlife species from Australia
Source: Parasit Vectors. 2020 Sep 5;13:447. doi: 10.1186/s13071-020-04325-6 (PMC7487544; doi:10.1186/s13071-020-04325-6)
Supplement: Supplementary file 4 — Additional file 4: Table S3. List of sequences used for phylogenetic analysis. [file 13071_2020_4325_MOESM4_ESM.docx]

**Additional file 4: Table S3.** List of sequences used for phylogenetic analysis.

| *Trypanosoma* species | Host | GenBank accession number | Animal group |
| --- | --- | --- | --- |
| *T. ranarum* | *Lithobates pipiens* | AF119810 | Amphibian |
| *T. neveulemairei* | *Rana* cf. *esculenta* | AF119809 | Amphibian |
| *T. mega* | *Amietophrynus regularis* | AJ223567 | Amphibian |
| *T. fallisi* | *Anaxyrus americanus* | AF119806 | Amphibian |
| *T. rotatorium* | *Lithobates catesbianus* | AJ009161 | Amphibian |
| *T. tungarae* | *Engystomops pustulosus* | KM406915 | Amphibian |
| *T. chattoni* | *Lithobates pipiens* | AF119807 | Amphibian |
| *Trypanosoma* sp. | *Scinax ruber* | EU021236 | Amphibian |
| *Trypanosoma* sp. | *Leptodactylus chaquensis* | EF457288 | Amphibian |
| *Trypanosoma* sp. | *Scinax hayii* | EU267075 | Amphibian |
| *Trypanosoma* sp. | *Rhinella schneideri* | EF457289 | Amphibian |
| *Trypanosoma* sp. | *Rhinella hoogmoedi* | EF457292 | Amphibian |
| *Trypanosoma* sp. | *Pristimantis* gr. *lacrimosus* | EU021224 | Amphibian |
| *Trypanosoma* sp. | *Leptodactylus latrans* | KP019970 | Amphibian |
| *Trypanosoma loricatum* | *Pelophylax ridibundus* | MH424306 | Amphibian |
| *T. therezieni* | *Chamaeleo brevicornis* | AJ223571 | Reptil |
| *T. clandestinus* | *Caiman yacare* | KP768285 | Reptil |
| *T. chelodinae* | *Emydura signata* | AF297086 | Reptil |
| *Trypanosoma* sp. | *Caiman crocodilus* | KP768293 | Reptil |
| *Trypanosoma* sp. | *Evandromyia infraspiosa* | EU021237 | Insect |
| *Trypanosoma* sp. | *Sciopemyia sordellii* | EU021243 | Insect |
| *Trypanosoma* sp. | *Sciopemyia servulolimai* | EU021241 | Insect |
| *Trypanosoma* sp. | *Sciopemyia sordellii* | EU021244 | Insect |
| *Trypanosoma* sp. | *Phlebotomus kazeruni* | AB520638 | Insect |
| *Trypanosoma theileri* | *Tabanus* sp. | AY971802 | Insect |
| *T. rangeli* | *Rhodnius brethesi* | EF071580 | Insect |
| *T. c. cruzi* | *Panstrongylus geniculatus* | AF288660 | Insect |
| *T. grayi* | *Glossina palpalis gambiensis* | AJ005278 | Insect |
| *T. triglae* | *Trigla lineata Gmelin* | U39584 | fish |
| *T. boissoni* | *Zanobatus atlanticus* | U39580 | fish |
| *T. murmanensis* | *Hippoglossus hippoglossus* | DQ016616 | fish |
| *T. ophiocephali* | *Channa argus (Cantor)* | EU185634 | fish |
| *T. granulosum* | *Anguilla anguilla (Linnaeus)* | AJ620552 | fish |
| *Trypanosoma* sp. | *Clarias angolensis Steindachner* | AJ620555 | fish |
| *T. pleuronectidium* | *Gadus morhua* | DQ016613 | fish |
| *T. cobitis* | *Hemiclepsis marginata* | AJ009143 | Annelida_Fish |
| *Trypanosoma* sp. | Leech | AJ009167 | Annelida |
| *T. conorhini* | *Rattus rattus* | XR_003828665 | Mammal |
| *T. minasense* | *Callithrix penicillata* | AJ012413 | Mammal |
| *T. irwini* | *Phascolarctos cinereus* | FJ649479 | Mammal |
| *T. cyclops* | *Wallaby* | AJ131958 | Mammal |
| *T.* sp. *ABF* | *Wallaby* | AJ620564.1 | Mammal |
| *T. noyesi* | *Trichosurus_vulpecula* | KX361179 | Mammal |
| *T. lewisi* | *Rattus* sp. | AJ223566 | Mammal |
| *T. copemani* | *Setonix brachyurus* | HQ267094 | Mammal |
| *T.* sp. *AB-2017* | *Phascolarctos cinereus* | KX786145 | Mammal |
| *T.* sp. | *Vombatus ursinus* | AJ009169 | Mammal |
| *T.* sp. | *Cervus dama* | AJ009165 | Mammal |
| *T. vespertilionis* | *Pipistrellus pipistrellus* | AJ009166 | Mammal |
| *T. theileri* | Buffalo | AY773674 | Mammal |
| *T. dionisii* | *Carollia perspicillata* | FJ001667 | Mammal |
| *T. terrestris* | *Tapirus terrestris* | KF586848 | Mammal |
| *T. gilletti* | *Phascolarctos cinereus* | GU966589 | Mammal |
| *Trypanosoma* sp. | Woylie | KC753533 | Mammal |
| *T. binneyi* | *Ornithorynchus anaticus Dun* | AJ620565 | Mammal |
| *Trypanosoma* sp*. 64* | *Trichosurus vulpecula* | JN315383 | Mammal |
| *T. rangeli* | *Platyrrinus lineatus* | EU867803 | Mammal |
| *T. c. cruzi* | *Didelphis marsupialis* | AF245382 | Mammal |
| *Trypanosoma* sp. | *Bettongia penicillata* | JN315392 | Mammal |
| *T. gennarii* | *Monodelphis domestica* | KT343360 | Mammal |
| *T. freitasi* | *Monodelphis brevicaudata* | MF401951 | Mammal |
| *T.*  *thomasbancrofti* | *Xanthomyza phrygia* | KT728395 | Avian |
| *T. avium* | *Corvus frugilegus* | U39578 | Avian |
| *T. corvi* | *Corvus frugilegus frugilegus* | AY461665 | Avian |
| *T. bennetti* | *Falco sparverius* | AJ223562 | Avian |
| *Leishmania chagasi* | *Canis lupus familiaris* | KF041780.1 | Outgroup |
| *Leishmania donovani* | *N/A* | XR_002966730 | Outgroup |
| *Leishmania mexicana* | *Homo sapiens* | KF041806 | Outgroup |
| *Cryptobia helicis* | *Helix pomatia* | AF208880 | Outgroup |
| *Bodo saltans* |  | AF208889 | Outgroup |
| *Leptomonas* | *Nabicula flavomarginata* | AF153043 | Outgroup |
| *Herpetomonas muscarum muscarum* |  | L18872 | Outgroup |
| *Phytomonas serpens* | *Lycopersicon* | U39577 | Outgroup |
