# Supplementary material for: Meta-transcriptomic identification of Trypanosoma spp. in native wildlife species from Australia
Source: Parasit Vectors. 2020 Sep 5;13:447. doi: 10.1186/s13071-020-04325-6 (PMC7487544; doi:10.1186/s13071-020-04325-6)
Supplement: Supplementary file 5 — Additional file 5: Table S4. Pairwise sequence identity among 18S rRNA sequences of avian trypanosomes belonging to genotypes 1–4 and the putative T. thomasbancrofti identified in this study. Genotype classification sensu Šlapeta et al. (2016) [41]. [file 13071_2020_4325_MOESM5_ESM.docx]

| Sequence genotype | G1_KT728373.1_2013_RegentHoneyeater | Vert11_DN10127_c0_g1_i1_len666 | G2_KT728395_2014RegentHoneyeater | G4_KT728402_2015RegentHoneyeater |
| --- | --- | --- | --- | --- |
| G1_KT728373.1_2013_RegentHoneyeater | - | 100 | 99.715 | 99.24 |
| Vert11_DN10127_c0_g1_i1_len666 | 100 | - | 99.7 | 98.348 |
| G2_KT728395_2014RegentHoneyeater | 99.715 | 99.7 | - | 99.145 |
| G4_KT728402_2015RegentHoneyeater | 99.24 | 98.348 | 99.145 | - |

**Additional file 5: Table S4.** Pairwise sequence identity among SSU 18S rRNA sequences of avian trypanosomes belonging to genotypes 1-4 and the putative *T. thomasbancrofti* identified in this study. Genotype classification sensu Šlapeta *et al*. 2016.
