# Supplementary material for: Meta-transcriptomic identification of Trypanosoma spp. in native wildlife species from Australia
Source: Parasit Vectors. 2020 Sep 5;13:447. doi: 10.1186/s13071-020-04325-6 (PMC7487544; doi:10.1186/s13071-020-04325-6)
Supplement: Supplementary file 6 — Additional file 6: Figure S2. Maximum likelihood tree showing phylogenetic relationships among trypanosomes within the aquatic clade based on the 18S rRNA gene. The trypanosome identified in Litoria fallax is indicated in blue. The hosts of trypanosomes are indicated with colour-coded tips. [file 13071_2020_4325_MOESM6_ESM.pdf]

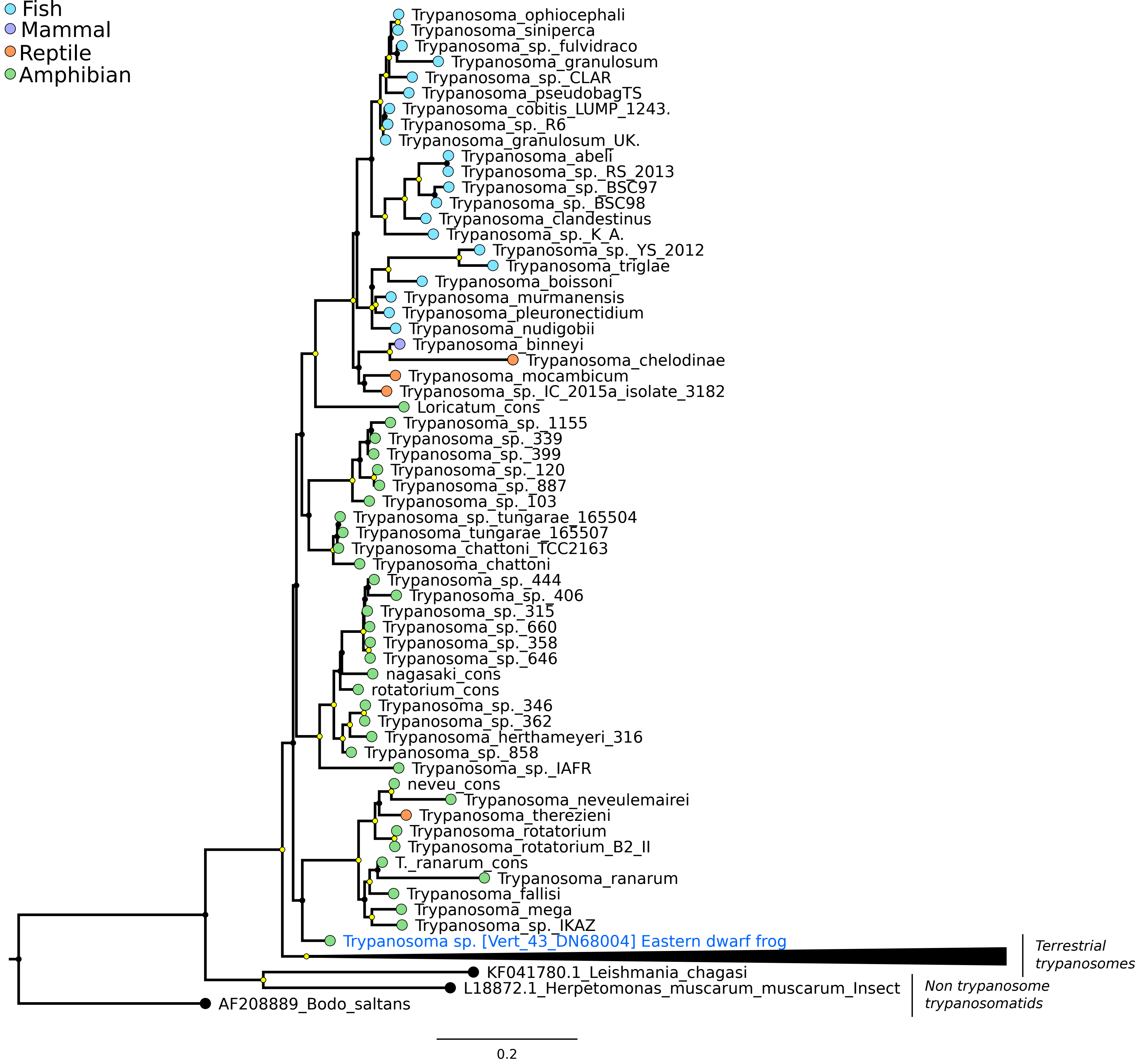

Additional file 6: Figure S2. Maximum likelihood tree showing phylogenetic relationships among trypanosomes within the aquatic clade based on the SSU 18S rRNA gene. The trypanosome identified in *Litoria fallax* is indicated in blue. The hosts of trypanosomes are indicated with colour-coded tips.
